# Dr. Walker, on Vaccination

**Published:** 1805-05-01

**Authors:** John Walker

**Affiliations:** Salisbury Square


### *To the Editors of the Medical and Physical Journal.*

GENTLEMEN,

A Director of the Royal Jennerian Society has, with some surprize and concern, called on me with the Star of yesterday, in which there is a paper on a subject which the writer considers a national one; a term however, which but very feebly designates its importance, as all the world is concerned in it. Ye will at once conclude that it must be that of which the discussion 'has increased, is increasing, and ought not to be diminished, till the small-pox is exterminated from the face of the earth.' Vacciolation is the theme; and Richard Dunning, Surgeon and Secretary to the Dock Jennerian Institution, (Plymouth) the ingeni ous and worthy author. Of all the advocates for the New Practice he has all along been, as ye well know, one of the most ardent to

> " Mark what yon father's uplift eyes bespeak,
> " What livelier transports on yon mother's cheek
> " Attest her joy."*

His ardour does not appear to be at all diminished, yet I fear that his notions, now committed to the wide world, may tend to diminish those parental raptures, and to lessen the confidence of the protected and their friends. That he never wished to shake their confidence, and that his own does not at all waver, is however evident, from the following unequivocal declaration. "I subscribe implicitly, at this time, a belief that the few subjects of incomplete vaccination who are liable to a secondary impression of variolous action (and the number of these who are liable, I believe, is very limited indeed) entirely possess all the exemptions and advantages attendant on favourable cases of the inoculated small-pox, exclusively of any risk of

 those

---

* The motto happily chosen for his pamphlet, reviewed in Vol. xii. of your Journal, p. 264.

afflicting and fatal consequences which so frequently follow and attach to the latter practice.

In your Review of his valuable pamphlet, ye have spoken of pages of it being principally theoretical, and some parts perfectly hypothetical. It appears to me to contain some very correct observations as well as ingenious conjecture. In the Star, even, he does continue to 'exercise his vein' for theorizing; and amid visionary ideas, he seems also to display considerable ingenuity, though I totally differ with him in the conclusion which he forms. "From much steady observation, from an extended correspondence, and from due reflection on all the circumstances which have arisen in the course of my attentions to this branch of medicine, I am led very confidently to advance an opinion, that entire and permanent resistance to variolous contagion, obtainable by vaccination, in some constitutions, depends in a great measure, *probably wholly depends,* on preserving *a vaccine vesicle unbroken during the process,* at least on taking care that it be not opened before that stage of it, when the areola is formed in all its characters. Indeed I would be understood to say, *where there is but one vesicle,* not before that is even beginning to decline."

From the constant demands for matter, at the Central House, I am daily obliged to break down the pocks at all stages, from their first formation till they have almost acquired their full dimensions. The matter collects again, the pocks bear their characteristic form, and the parents are assured that their children are perfectly protected. But, among the letters which I have from time to time been favoured with from the Discoverer, I can lay my hands upon one which will afford a good extract to this very point.

"I destroyed the pustule completely on the eighth day, before the areola began to form, by the application of a bit of caustic; but on putting my patients to a test; one, at the expiration of a year; the other, at two years, they were found secure. Again, Is not the virus contained in separate cells like the essential oil in the rind of an orange? How then can puncturing the pustule at any period destroy its influence on the constitution. Once more, Do we not see the pustule resume its character after it has been opened on an early day and exhausted, or nearly so."

Your's, respectfully,

*Salisbury Square,* 16, *iv,* 1805. JOHN WALKER.

